# A review focuses on a neglected and controversial component of SCI: myelin debris

**DOI:** 10.3389/fimmu.2024.1436031

**Published:** 2024-11-22

**Authors:** Yuchen Zhou, Tao Xu, Yiyan Zhou, Wei Han, Zhengchao Wu, Changwei Yang, Xiaoqing Chen

**Affiliations:** ^1^ Department of Spine Surgery, Affiliated Hospital of Nantong University, Nantong, China; ^2^ Medical School of Nantong University, Nantong, China; ^3^ Department of Orthopedics, Yancheng Dafeng People's Hospital, Yancheng, China; ^4^ Institute of Regenerative Biology and Medicine, Helmholtz Zentrum München, Munich, Germany

**Keywords:** spinal cord injury, myelin debris, myelin-associated inhibitors, axon, cholesterol, foam cell

## Abstract

Myelin sheath, as the multilayer dense structure enclosing axons in humans and other higher organisms, may rupture due to various injury factors after spinal cord injury, thus producing myelin debris. The myelin debris contains a variety of myelin-associated inhibitors (MAIs) and lipid, all inhibiting the repair after spinal cord injury. Through summary and analysis, the present authors found that the inhibition of myelin debris can be mainly divided into two categories: firstly, the direct inhibition mediated by MAIs; secondly, the indirect inhibition mediated by lipid such as cholesterol. It is worth noting that phagocytes are required in the latter indirect inhibition, such as professional phagocytes (macrophages et al.) and non-professional phagocytes (astrocytes et al.). Moreover, complement and the immune system also participate in the phagocytosis of myelin debris, working together with phagocytes to aggravate spinal cord injury. In conclusion, this paper focuses on the direct and indirect effects of myelin debris on spinal cord injury, aiming to provide new inspiration and reflection for the basic research of spinal cord injury and the conception of related treatment.

## Highlights

- The authors systematically concluded almost all the impacts of myelin debris after spinal cord injury, divided into two main aspects: direct and indirect impacts.
- The authors summarized the related studies of eight myelin-related inhibitors which induce the collapse of axon growth cone, including their structures, receptors and downstream signaling pathways.
- The authors introduced the origination, negative effects and countermeasures of "foam cells" in SCI in detail, which is, to an extent, more comprehensive than the previous studies.
- The authors summarized and discussed about three different views on the actual role of myelin debris in axon regeneration in spinal cord injury.
- It is worth mentioning the latest review related to foam cells in SCI [113]. This article presents most of the views similar to ours, especially on the description of the formation mechanism and solution to reducing foam cells. However, there still exists room for improvement in the summary of foam cells. The specifics are as follows:

- In view of the formation mechanism of foam cells, only two ways are provided to reduce foam cells in the latest review: reducing lipid intake and increasing intracellular lipid transport. However, our paper proposed another two schemes: promoting apoptosis of foam cells and promoting lipophagy of foam cells.
- The latest review focuses on the formation mechanism of foam cells and the above two measures to reduce foam cells, while pays less attention to the pathophysiological effects of foam cells on SCI. In contrast, we have discussed the pathophysiological effects in detail, including aggravating inflammation, promoting scar, hindering the myelination of newborn axons, and hindering the differentiation of neural stem cells into neurons.
- One of the main opinions of the latest paper is the perceived pro-inflammatory effects of foam cells. Instead, we discuss the potential anti-inflammatory effects of foam cells from another angle.

## 1 Introduction

Multiple layered structures wrap nerve fibers in the nervous system of higher organisms, which is called myelin sheath. Myelin sheath not only protects the axons, but also greatly speeds up the transmission speed and efficiency of electrical signals, so myelin sheath is crucial to the nervous system (2, 3). The whole nervous system can be further divided into the central nervous system (CNS) and the peripheral nervous system (PNS). The myelin sheath in CNS originates from oligodendrocytes, while in PNS, the role of myelin sheath formation is played by Schwann cells (4, 5). For one thing, these two types of cells—oligodendrocytes and Schwann cells form myelin sheath; for another, they constantly communicate with axons to provide protection and nutritional support, which is indispensable for the survival and integrity of axons (6). After spinal cord injury (SCI), the communication between oligodendrocytes and axons is interrupted due to the primary injury caused by immediate mechanical impact and the subsequent secondary injury caused by long-term bleeding, ischemia, free radical, immune inflammation et al., giving rise to the formation of myelin debris at the injury site. The myelin debris exists at the injury site for a long time, which remains one year after injury (6–10). Interestingly, unlike intact myelin sheath, fragmented myelin sheath (myelin debris) can worsen the microenvironment of SCI, hinder neuronal regeneration, and ultimately affect the recovery of sensory and/or motor function (11). Since Berry et al. ‘s initial proposal of the inhibitory effect of myelin debris, the present authors have summarized the inhibitory effect into two main aspects: the direct effect from inhibitory substances in myelin debris and the indirect inhibitory effect from the phagocytosis of lipid in myelin debris (12). Some scholars believe that there are two main obstacles to nerve regeneration after SCI: inhibition of myelin debris and glial scar; the former plays the dominant role before scar maturation (11). Therefore, it is essential for SCI repair to eliminate the devastating effects of myelin debris, which requires researchers to firstly identify the specific effects of myelin debris on the pathological process of SCI. However, few scholars have systematically sorted out and summarized about the effects yet, especially one of the indirect effects of myelin debris– when macrophages engulf large amounts of lipid-rich myelin debris, they will exhibit the same characteristic as foam cells in atherosclerosis because they fail to catabolize endocytic myelin debris for their poor lipid metabolism capacity. In view of this gap, this paper aims to explore the direct and indirect effects of myelin debris on SCI, in order to provide new references for basic research related to SCI and the recovery of sensory and/or motor function (**Table 1**).

**Table 1:** The summary of the inhibitory effects of myelin debris.

The direct inhibitory effect of myelin debris	On neuron	Inducing the collapse of axon growth through eight myelin-related inhibitors: Nogo, Mag, Omgp, Netrin-1, Ephrin-B3, Versican, Brivican, Sema-4D	Nogo: since 2000, Grandpre et al. Mag: since 1996, Deb Ellard et al. Omgp: since 2005, Huang et al. Netrin-1: since 2008, Low et al. Versican/Brivican: since 1999, Niederost et al. Sema4D: since 2002, Swiercz et al. Ephrin-B3: since 2004, Goldshmit et al.
	On oligodendrocyte	Hindering the maturation and differentiation of oligodendrocytes	Since 1999, Robinson et al.
The indirect inhibitory effect of myelin debris	Phagocytosis of microglia/macrophages	Microglia and macrophages will secrete more inflammatory factors after engulfing myelin debris, of which macrophages can also develop into foam cells, hindering the recovery of spinal cord injury by magnifying inflammation, down-regulating phagocytosis and aggravating scar	Since 2006, Landry et al. 2022, Chen et al. 2024, Xue et al.
	Phagocytosis of astrocytes	After phagocytosis of myelin debris, astrocytes can exacerbate local inflammation, enhance their own proliferation and aggravate glial scars	Since 2006, Landry et al. 2021, Chen et al.
	Phagocytosis of endothelial cells	The phagocytosis of myelin debris by endothelial cells can exacerbate local inflammation and aggravate fibrous scars	Since 2011, Stammers et al. 2019, Zhou et al.
	Complements and immunity	After myelin debris binds to the antibody, it undergoes opsonophagocytosis conducted by complement system, which further damages the intact myelin sheath, enhances inflammation and damages surviving neurons	Since 1997, Johns et al.

## 2 Direct inhibitory effect of myelin debris

The initial understanding of the direct inhibitory effect of myelin debris began with myelin membrane binding proteins (weighing 35kDa, 250kDa). Schwab and Thoenen et al. cultured sympathetic neurons in a microenvironment composed of sciatic nerve (kind of peripheral nerve) and optic nerve (kind of central nerve) respectively. They found that the sympathetic neurons cultured with the sciatic nerve had strong vitality and correspondingly a large number of axons while those cultured with the optic nerve showed poor vitality with almost no axons observed. This opposite phenomenon promotes the exploration of inhibitors in CNS (13). Over the years, multiple inhibitory substances in myelin debris have been identified, and the mechanisms are becoming clear: dependent on receptor-ligand interactions and activation of downstream signaling pathways.

### 2.1 Direct inhibition of myelin debris on axons

**Figure 1:**
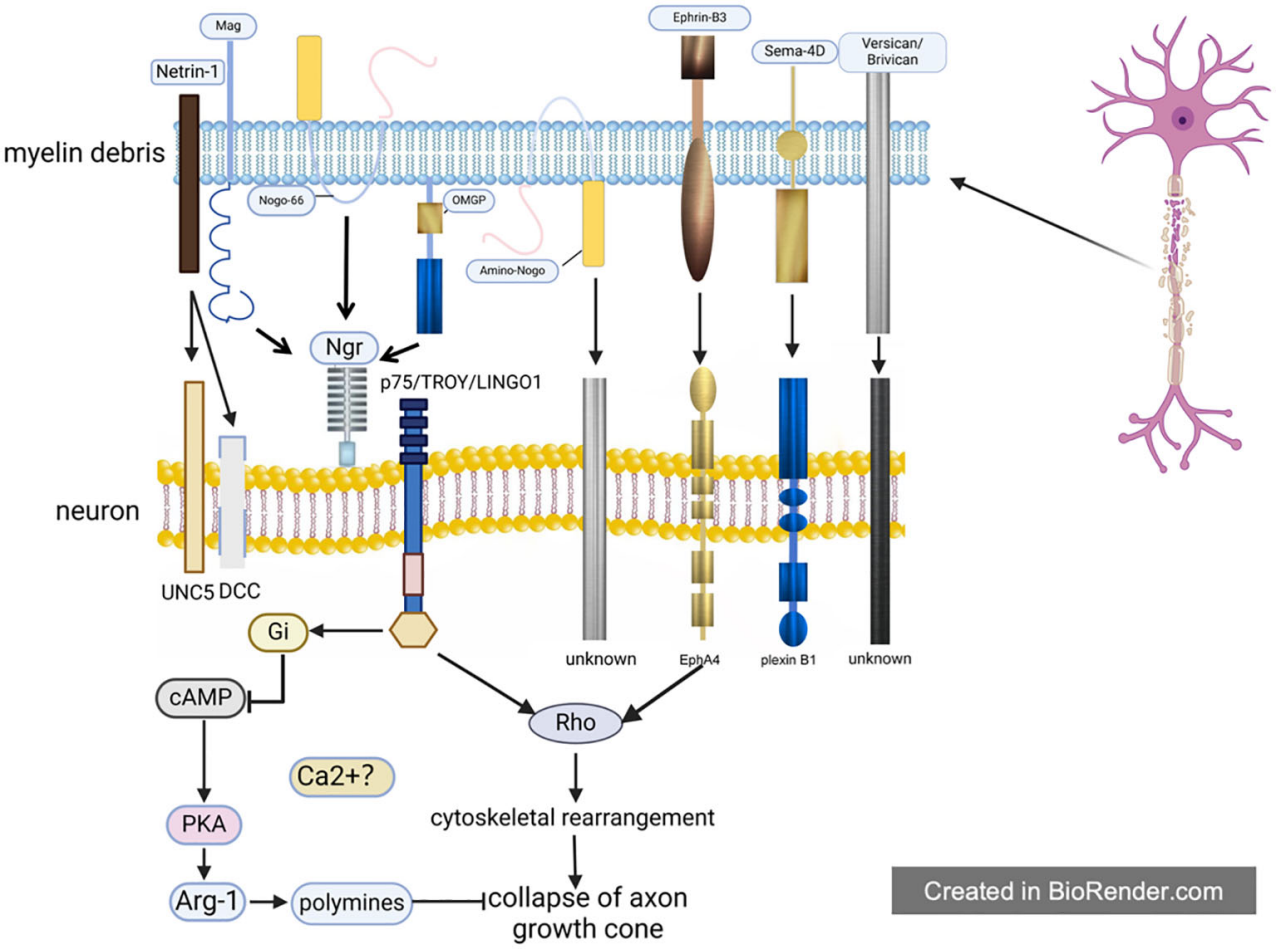
The direct inhibitory effect of myelin debris. After spinal cord injury, there exist large amounts of myelin debris at the lesion site, which will inhibit the growth of axon cones through 8 MAIs inside. The function of MAIs depends on their receptors, among which Ngr is shared by Nogo, Mag and Ompg. Moreover, Ngr has been most studied by now, whose downstream pathways are basically clear. However, it is still unknown about the mechanisms involved in most of these receptors, even as well as the MAIs receptors such as the receptor of Ephrin-B3 et al.

As for MAIs, some scholars only briefly mentioned several different substances in some studies without discussion in detail. Through summary, the present authors found that a total of eight MAIs have been explored up to now: Nogo; Mag; Omgp; Netrin-1; Versican; Brivican; Transmembrane signaling protein 4D; Ephrin-B3, among which Nogo, Mag and Omgp were three kinds of classic MAIs with more attention (14). After SCI, these eight MAIs exert their inhibitory effects on axons independently or in group through receptor-ligand interactions (**Figure 1**).

#### 2.1.1 MAIs

##### 2.1.1.1 Nogo

Besides Schwab and Thoenen, Schwab and Caroni et al. also observed a phenomenon regarding the inhibitory effect of optic nerve on the growth of axons and fibroblasts. After a series of rigorous researches, the team suggested that the inhibition of myelin debris could be attributed to membrane binding proteins with molecular weights of 35kDa and 250kDa (15). With the deepening of research in this field, later scholars found that the antibodies of membrane binding proteins above can bind a total of three similar antigens, including 35kDa and 250kDa membrane binding proteins, and Schwab uniformly named them Nogo (11, 16–18). There are three isoforms of Nogo totally, namely type A, type B and type C, among which Nogo-A is the most abundant in myelin sheath. Moreover, Nogo is also a member of the reticulin family, which usually contains proteins associated with the ER (18–20). Nogo-A contains at least two axon inhibitory domains: an N-terminal domain (known as amino-Nogo) and a 66 amino acid rings (known as Nogo-66) located between two transmembrane structures. Notably, amino-Nogo, exclusive to Nogo-A, is distributed both inside and outside the nervous system and can also interact with non-nerve cells. However, Nogo-66, a domain shared by all three isoforms of Nogo, exists only in CNS and only acts on neurons. After SCI, when intact myelin sheath or myelinated oligodendrocytes are destroyed, these inhibitory domains are exposed to the microenvironment, leading to the failure of axon regeneration (11, 18, 21).

##### 2.1.1.2 Mag

Compared with Nogo, Mag is actually the first MAI identified by human, which induces the collapse of axon growth cone (22–24). As a member of the immunoglobulin family, Mag contains five extracellular Ig-like domains, the first four Ig-like domains of which are homologous to other sialic acid binding Ig-like domains (Siglec). Therefore, Mag is also a member of the Siglec family (25, 26). However, Kelm et al. pointed out that the combination of Mag and sialic acid may only enhance May’s inhibition on axon growth cone but is not necessary for its axon-inhibiting effect. Therefore, it is still unclear about the specific role of sialic acid in Mag inhibiting axon growth, where further studies are needed (27). Different from the view that Mag only has inhibitory effect on axon growth, several scholars have found that Mag has a dual effect on axon growth— both promoting and inhibiting axon growth. This kind of dual effect depends on the developmental stage of the neurons. In simple terms, Mag promotes axon growth in embryos or newborns while it inhibits axon growth in adults, its function occurring at or shortly after birth (24, 28). It is worth mentioning that regarding the mechanism of this axon-promoting effect, some scholars have proposed that Mag can protect neurons from acute toxic substances through a ganglioside dependent way. However, it still needs further investigation how Mag promotes nerve axon growth, whether the receptor that mediates its inhibitory effect also has promoting effect and why Mag transforms from promoting to inhibiting axon growth (29).

##### 2.1.1.3 Omgp

Omgp (oligodendrocyte myelin-associated glycoprotein) is the third MAI found to suppress the axon growth cones. Functionally, Huang et al. initially reported the axonal inhibition of Omgp. Specifically, Omgp is expressed in mature oligodendrocytes and suppresses the germination of abnormal lateral branch of Ranvier tubercles. Later, Omgp was identified by Wang et al. as a member of MAIs after Nogo and Mag (30–32). Structurally, Omgp is a glycosyl phosphatidylinositol (GPI) linked protein that contains a leucine-rich repeating domain. Despite its name suggesting a specific expression pattern, Omgp is not only highly expressed in oligodendrocytes, but also significantly expressed horizontally in various neurons (32, 33).

##### 2.1.1.4 Netrin-1

Although previous researches on the three classic MAIs have been conducted in depth, it seems that it is still difficult to promote SCI recovery by only intervening on the three proteins above. Therefore, the question whether there exist other unknown MAIs was provoked (20, 34–37). Low et al. found that in addition to the three classical MAIs, axon growth induction-1 (Netrin-1) was also contained in myelin debris. This protein induces axon growth cone collapse both *in vivo* and *in vitro*, and the growth of axon was promoted after the neutralization of Netrin-1. Moreover, further studies showed that, *in vivo*, Netrin-1 is primarily located next to the tubercles of Ranvier, similarly located to that of two other MAIs, Omgp and Mag. However, different from Omgp and Mag, Netrin-1 is a secretory ligand rather than a transmembrane protein (14, 38), which means that it may play a different role in intercellular communication. In addition, like Mag, Netrin-1 has a dual function, whose dual function is achieved by interacting with two receptors on neurons with opposite functions yet. However, the fact is that in adults, receptors mediating axon inhibition predominate in number, which means that their axon growth will be inhibited by Netrin-1 after SCI (14).

##### 2.1.1.5 Versican, Brivican

Not all MAIs are composed of proteins, such as Versican and Brivican. Chondroitin sulfate proteoglycan (CS-PG) is the most abundant and widely distributed glycosaminoglycan in the body. After SCI, CS-PGs expression is up-regulated. On one hand, these increased CS-PGs participate in the formation of glial scars, which not only can cut off the inflammatory microenvironment but also impedes the connection of regenerated nerves at both ends of the injury. On the other hand, there exist abundant and harmful chemical substances in CS-PGs, impeding nerve regeneration. In general, CS-PG is negative on nerve growth (39–41). It is generally accepted that CS-PG is mostly produced by astrocytes, but Asher et al. pointed out that Versican and Brivican, though classified as CS-PG, originate from oligodendrocytes and their number increase by the effect of TGF-β after SCI, which inhibits axon growth. Quantitative analysis of the Versican 7th day after SCI shows that the number of Versican increased by 2 to 3 times compared with that before injury (42, 43). Similar to that of several mentioned MAIs, the expression of Versican and Brivican has some certain spatial-temporal characteristics: Versican is only expressed in mature oligodendrocytes or myelinated oligodendrocytes, which is closely related to the formation of myelin sheath. In addition, Versican belongs to the hyaluronic acid -bound CS-PG polyproteoglycan family, which was originally identified in the adult CNS as a glial hyaluronic acid-binding protein (GHAP), whose N-termini were identified as domains that exert binding functions. Therefore, the release of Versican requires the involvement of hyaluronidase (43–47). The special composition of Versican and Brivican suggests that the two may be dealt with from the perspective of CS-PG and hyaluronic acid, as well as the relationship between CS-PG and astrocytes. Moreover, it is also a question worthy of attention whether glial scar and axon inhibition mediated by Versican and Brivican can be solved simultaneously from the perspective of CS-PG.

##### 2.1.1.6 Sema4D

Sema4D, also known as CD100, is a homodimer transmembrane protein with a molecular weight of 150 kDa, originally discovered in the immune system. Sema4D is expressed on most hematopoietic cells and can also activate B cells or T cells. As understanding on Sema4D deepens, scholars have gradually discovered its new functions in CNS (48–51). After co-culturing oligodendrocytes with neurons, Moreau-Fauvarque et al. pointed out that Sema-4D, a new axon inhibitor, existed in myelin debris. What’s more, Sema4D was found to be expressed in oligodendrocytes throughout CNS, and its peak value was reached during myelination. By contrast, the team found that the number of Sema-4D in oligodendrocytes increased briefly at 8th day after SCI and recovered to the pre-injury level at 30th days after SCI. Moreover, the number of Sema4D is also affected by the differentiation state of oligodendrocytes. Moreau-Fauvarque et al. found that Sema4D can be expressed only in mature oligodendrocytes or myelinated oligodendrocytes at both gene and protein levels, with the presence of Sema4D rarely detected in the early differentiation stage of oligodendrocytes. Besides such temporal characteristics, Sema4D expression also has certain spatial characteristics given that Schwann cells and oligodendrocytes have something in common in many aspects. Moreau-Fauvarque et al. explored whether Sema4D exists in Schwann cells and found that Sema4D was not expressed in Schwann cells (52). In conclusion, Sema4D, as a member of MAIs, can promote the collapse of axon growth cones, and it exists only in the mature oligodendrocytes with a short-term increase in number after SCI.

##### 2.1.1.7 Ephrin-B3

Nogo, Mag, and Omgp share the same receptor, Ngr (mentioned below). In theory, blocking this receptor could profoundly alleviate axon growth inhibition. However, the results were not satisfactory. Then, Ephrin-B3 was identified as one of MAIs (53). Through gene sequencing and western blot, Benson et al. confirmed that Ephrin-B was only expressed on mature oligodendrocytes or myelinated oligodendrocytes, which was consistent with other MAIs (54, 55). Despite functional similarities among Ephrin-B3 and other MAIs, Ephrin-B3 exhibited stronger inhibitory activity. Benson et al. co-cultured neurons with three classical MAIs and Ephrin-B3 at the same concentration *in vitro*, and the results showed that the number of neurons in Ephrin-B3 group was significantly lower than that in other groups. They also found that the combined closure of the receptor of Ephrin-B3 and Ngr had a better effect on axon growth, compared to the closure of Ngr alone. In addition to Ephrin-B3, Ephrin also has other subtypes. To further explore whether these subtypes exist in myelin debris and can inhibit axon growth like Ephrin-B3, Benson et al. detected Ephrin A1-A5 and B1-B3. The results showed that only Ephrin-B3 was expressed in the myelin sheath or myelin debris in CNS, promoting the collapse of axon growth cone (55).

#### 2.1.2 Receptors of MAIs

Following the understanding of the MAIs mentioned above, it is important to further explore how they mediate the collapse of axon growth cone, which is greatly enlightening to the treatment of SCI. Nowadays, it has been discovered and identified about most of the mechanisms and several key signaling pathways with their effector molecules. Next, the present authors will elaborate on these receptors and their downstream signaling molecules.

##### 2.1.2.1 Co-receptor of Nogo, Mag and Omgp

Despite structural differences, Nogo, Mag and Omgp have been confirmed by multiple experiments that they act on the same receptor, Ngr. In this process, p75 neurotrophic receptor (p75^NTR^) assists transmembrane signal transduction as a co-receptor, enabling the axon inhibitory effect of Ngr to be effectively realized (11, 56, 57). Ngr is expressed on the surface of different neurons, and its structure is composed of glycosylphosphatidylinositol (GPI) linker proteins with a molecular weight of 85kDa. Moreover, there exist multiple leucine-rich repeat domains in Ngr, which are followed by a unique C-terminal sequence. The initial understanding of Ngr began with the study on Nogo-66. For example, Strittmatter et al. found that some membrane proteins could be bound by Nogo, so they named them Nogo receptor (Ngr) and clearly pointed out that the connection between Nogo-66 and Ngr is necessary for Nogo to function (21). However, amino-Nogo has a weak affinity with Ngr, and only blocking Ngr did not effectively improve its inhibitory effect. In addition, amino-Nogo is proved to be widely distributed, both inside and outside the nervous system. Though the physical characteristics of amino-Nogo are partly mastered, real receptors and its downstream signaling pathway of the amino-Nogo are still unclear, which still need further study.

Since Ngr is a GPI linker protein which is only located on the surface of the cell membrane, it is difficult for Ngr to independently perform the transmembrane signal transduction, suggesting that there may exist auxiliary receptors to assist Ngr in transmitting inhibitory signals (58). In fact, prior to the discovery of Ngr, it had been reported that p75^NTR^ was a common receptor of Nogo, Mag and Omgp, but the direct interaction between Mag and p75^NTR^ failed to be confirmed (59). With the deepening of research, scholars gradually realized that p75^NTR^ belongs to the member of the tumor necrosis factor receptor (TNFR) family, which plays the role of Ngr coreceptor and certainly does not directly contact with Mag et al. Because of p75^NTR^ blocked, the vitality of three classical MAIs can be successfully inhibited. However, p75^NTR^ is not expressed on the surface of most neurons as thought, implying that there may exist other unknown co-receptors. Consequently, TROY and LINGO1, two other TNFR family members, were later identified as co-receptors of Ngr. Blocking these two co-receptors can also alleviate the axon inhibition of p75^NTR^. It’s worth mentioning that the structure of Ngr provides important clues how these three classic MAIs function in time and space: The binding domains of the three are proved to exist simultaneously on Ngr; Nogo, Mag and Omgp may act on Ngr at the same time or work at different times to inhibit axon growth (11, 60–63).

The downstream pathway of Ngr-P75^NTR^/TROY/LINGO1 has been identified, and several scholars have demonstrated that Ngr-P75^NTR^/TROY/LINGO1 can transmit inhibitory signals by activating Rho, followed by the change of cytoskeleton, which ultimately resulted in the collapse of axon growth cone. The inactivation of Rho caused by toxin C3 can counteract the inhibitory effect of Nogo et al., promoting extensive nerve regeneration and functional recovery in mice with SCI (64–66). Like most signaling cascades affecting the cytoskeleton, calcium has also been found to be likely involved in the downstream signaling pathway of Ngr-P75^NTR^/TROY/LINGO1: myelin debris has been shown to induce an increase of calcium in neurons, and axon growth cone collapse induced by Nogo et al. is calcium-dependent. However, it remains unknown that what role calcium plays in the signaling pathway and where it originates (67). In addition, some scholars have found that there are new signaling molecules in the interaction between Mag and Ngr -P75^NTR^/TROY/LINGO1 after SCI: cAMP. For one thing, cAMP was found to directly inhibit Rho and prevent Rho from affecting cytoskeleton; for another, it could activate PKA, and then increase the number of arginase I (Arg-1), followed by the increasing amount of dopamine. Finally, the collapse of axon growth cone was induced under the influence of dopamine by affecting the cytoskeleton and blocking the inward rectifier potassium channel. Likewise, even some experiments have shown that only up-regulation of cAMP is sufficient to alleviate the axon growth inhibition caused by Ngr -P75^NTR^/TROY/LINGO1. Unfortunately, cAMP was inhibited by Nogo et al. when they interact with Ngr-P75^NTR/^TROY/LINGO1 after SCI, which enhanced the axon inhibition of Nogo et al., forming a vicious cycle (53, 68–70). Filbin et al. (11) found that although newborn neurons have Ngr, they are not sensitive to MAIs such as Nogo et al., which may be explained by the fact that newborn neurons contain a large amount of cAMP compared with old ones. Therefore, the team proposed that the “MAIs” actually might not be axon inhibitors, the collapse of axonal growth cones caused by which is simply because the number of cAMP affects the response of neurons to MAIs. That is to say, cAMP changes the intrinsic state of neurons. Considering the low responsiveness of newborn neurons to MAIs, the present authors believe that the complex role of MAIs needs reconsideration as well as the hypothesis of cAMP with the state of neuron.

Although certain neural repair has been achieved by blocking Ngr, some scholars have pointed out that blocking Ngr alone cannot significantly alleviate the axon inhibition mediated by Nogo et al. after SCI. Therefore, new receptor of Nogo on the surface of neurons was found, which was respectively called pairing immunoglobulin-like receptor B (PirB) in mouse, and human leukocyte immunoglobulin-like receptor B2(LilrB2) in human. Atwal et al. further confirmed that these receptors have a higher affinity for MAIs such as Nogo than Ngr. In addition, Atwal also pointed out that the action period of Ngr and PirB/LileB2 is different— Ngr mainly mediates acute axon inhibition; PirB/LileB2 mainly mediate axon inhibition when MAIs were present for a long time. Therefore, the combined closure of these receptors can largely alleviate the MAIs-induced axon growth cone collapse (37). Regarding the understanding of PirB/LileB2 receptor, though some progress, it is still unclear about the downstream signaling pathways and signaling molecules of the two. Future studies are suggested to focus on this field in order to reduce the axon inhibition of myelin debris more efficiently.

##### 2.1.2.2 Netrin-1, Sema4D and Ephrin-B3-related receptors

As mentioned above, the dual effect of Netrin-1 on axon growth cones is attributed to its ability to interact with two receptors with opposite functions: UNC5 and DCC. UNC5 can induce the collapse of axon growth cone while DCC does the reverse. From the perspective of space-time, UNC5 and DCC were mainly distributed in mature or myelinated oligodendrocytes in CNS. In the development of human, DCC was dominant in juvenile, while UNC5 was dominant in adult (14, 71–73). Similarly, Sema4D works through two different receptors: plexinB1 and CD72. Interestingly, plexinB1 and CD72 also have spatial-temporal characteristics: the number of plexinB1 gradually increased in few parts in CNS like spinal cord in the process of growth, but CD72 is widely expressed in CNS after birth. Consequently, it is generally believed that the axon inhibition of Sema4D may be mainly mediated by CD72 (52, 74, 75). Regarding Ephrin-B3-related receptors, Benson et al. demonstrated that the receptor of Ephrin-B3 was EphA4, which was up-regulated on the surface of neurons after SCI, and the axon inhibition mediated by Ephrin-B3-EphA4 was equivalent to the sum of Ngr-p75^NTR^/TROY/LINGO1 (55). Regarding the downstream signaling molecules of EphA4, some scholars have found that EphA4, like P75^NTR^/TROY/LINGO1, can also activate Rho, indicating that their signaling pathways may overlap (76). Thus, nerve regeneration observed through inhibition of Rho or Rho kinase may be attributed to the obstruction of EphA4 and/or p75^NTR^/TROY/LINGO1. However, there still exists a gap in the field of receptor of Versican and Brivican on neurons yet, which needs further investigation.

### 2.2 Direct inhibition of myelin debris on oligodendrocytes

The myelin debris, as a part of mature/myelinated oligodendrocytes, reacts against the oligodendrocytes, but only affects the differentiation rather than the recruitment of oligodendrocytes (i.e. affecting quality rather than quantity). However, the myelination of axon in CNS requires a large number of differentiated oligodendrocytes, so myelin debris can further hinder SCI recovery by interfering with the normal physiological function of oligodendrocytes. Kotter et al. added equal amounts of myelin debris, liver cell membranes and PBS into the spinal cord to compare their effects on oligodendrocytes respectively. They found that the neurons in myelin debris group had significantly lower levels of myelin basic protein [MBP, a protein that can be used to label mature/myelinated oligodendrocytes (77)] than MBP in other two groups, while the liver cell membranes group had slightly more MBP and the PBS group had the most, confirming that myelin debris affects the number of mature/myelinated oligodendrocytes. What’s more, two main factors could contribute to the small number of mature/myelinated oligodendrocytes observed, respectively the actual number of oligodendrocytes in the spinal cord and the maturation of existing oligodendrocyte precursor cells (OPC). By quantitative analysis, they found similar oligodendrocyte density among the three groups, indicating that myelin debris mediates the disorders of differentiation of oligodendrocytes without affecting their numbers. Although the liver cell membranes were also observed to hinder the differentiation of oligodendrocytes to a degree, the team explained that this kind of effect was still indirect and mainly caused by the inhibition of myelin debris. The liver cell membrane aggravated the phagocytosis burden of macrophages, resulting in a consequent reduced phagocytosis of myelin debris, so that myelin debris accumulated at the lesion site, whose inhibitory effect had been demonstrated (77–79). Robinson et al. also found that myelin debris influences the differentiation of OPC, and this kind of inhibition is concentration dependent. After comparing myelin debris in CNS with counterpart in PNS or other cell membranes, scholars found that this inhibitory effect on OPC was limited to myelin debris in CNS (80). Although the inhibitory effect of myelin debris on oligodendrocytes has been found, the internal mechanism is still unknown. The present authors believe that it may also be because of the involvement of MAIs, but it still needs further exploration about the kind of MAIs, its receptors and downstream signal pathways.

The direct inhibitory effects of MAIs on axons and oligodendrocytes have been discussed thereinbefore, which impede nerve regeneration through obstruct axon regeneration and axon remyelination. Through summary and analysis, the present authors found that there are some commonalities in the inhibitory effects: Whether or not MAIs have dual functions, they all show inhibitory effects on axonal growth cones after SCI in adult; meanwhile it is believed that it may be related to the content of cAMP in neurons, which may account for the reason that the prognosis of patients with SCI deteriorates gradually with physical maturity. Despite considerable progress on MAIs, there still exist many questions to be solved, such as the relationship between calcium and signal transduction of Ngr. Calcium is widely recognized for its involvement in a variety of biological processes. However, it has not been clearly identified about the source of calcium, as well as the upstream effector molecules and downstream regulatory targets involved in axon inhibition mediated by Ngr,. In addition, although the three classical MAIs were believed to play a major role in axon growth cone inhibition, it now seems that the situation may not be the case, and the difference in experimental methods may lead to the reduction of the proportion of other MAIs. Therefore, the specific proportion of these eight MAIs in axon growth cone inhibition deserves further exploration as well as calcium.

## 3 Indirect inhibition of myelin debris

**Figure 2:**
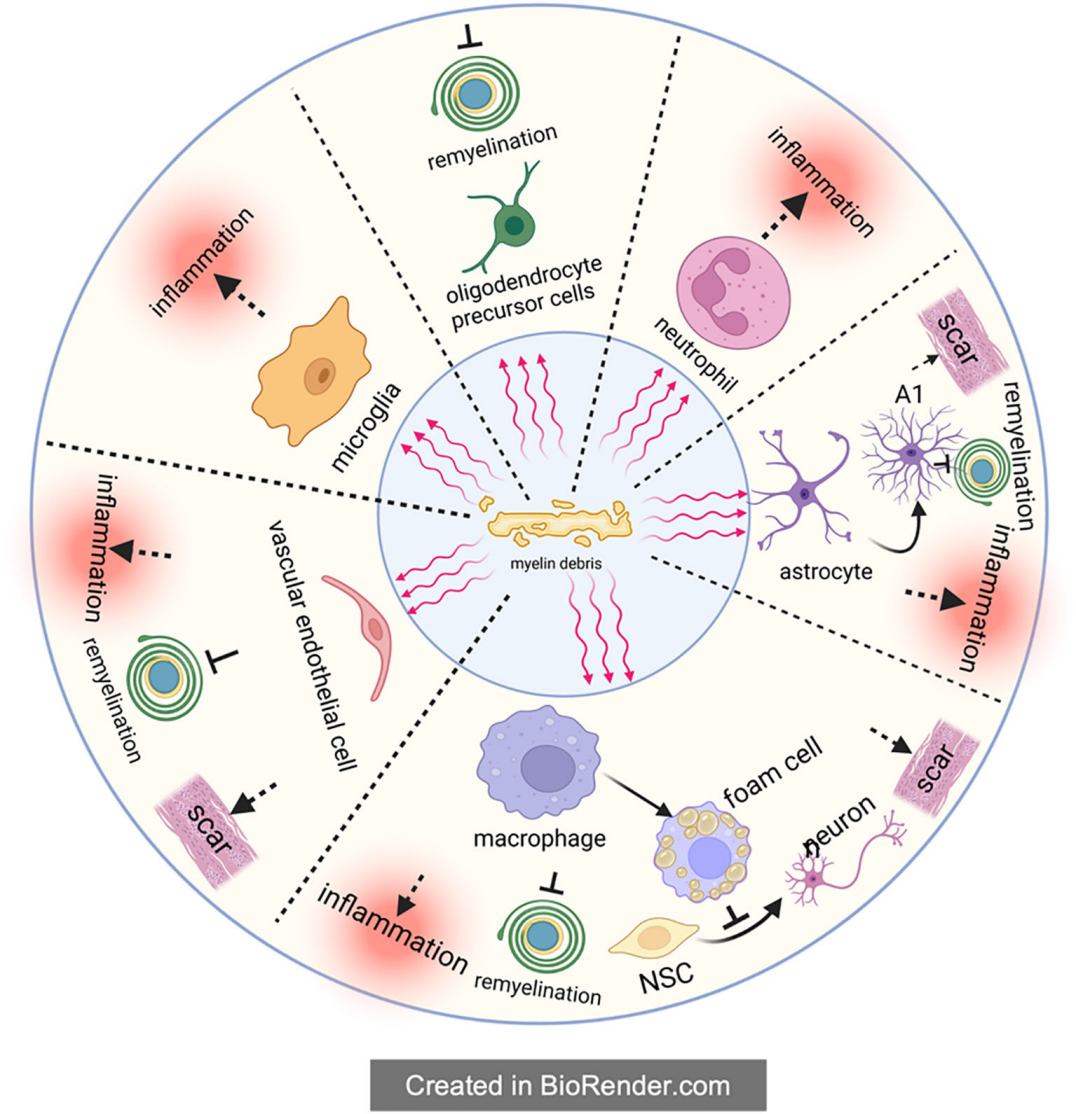
The indirect inhibitory effect of myelin debris. Upon the form of myelin debris in the SCI microenvironment, it will be engulfed by phagocytes to maintain the homeostasis of microenvironment. Theoretically, phagocytosis of myelin debris by phagocytes is beneficial to SCI recovery, but in fact these phagocytes will accordingly change their own state and then exert multiple negative effects on SCI recovery, such as amplifying inflammation, preventing remyelination, impeding the differentiation from NSC to neuron and aggravating the scar. It is worthy of noting that macrophages will transform into foam cells after engulfing myelin debris; foam cells in SCI, partly like in atherosclerosis, exacerbate SCI severely.

After SCI, myelin debris will be compensatively engulfed to avoid its negative effects, but this process also exert inhibition on nerve regeneration, which is called the indirect inhibition mediated by myelin debris. Unlike the direct inhibition of myelin debris on axon regeneration and remyelination, when dealing with myelin debris, these phagocytes will therefore up-regulate the expression of inflammatory pathways (such as NF-κB), release inflammasomes, and then result in the formation of inflammatory storm in the microenvironment, accelerating the demyelination and apoptosis of remaining neurons and eventually leading to neurological impairment (81, 82) (**Figure 2**).

In conclusion, the present authors hold that the indirect inhibition is mainly caused by the following three aspects: (1) phagocytosis of professional phagocytic cells represented by microglia/macrophages; (2) phagocytosis of non-professional phagocytes like vascular endothelial cells; (3) the involvement of complement and immune system. On this basis, the present authors further give a more detailed and comprehensive description of the inhibition myelin debris, especially the “foam cells” in SCI.

### 3.1 Microglia/macrophages

#### 3.1.1 Formation of foam cells

**Figure 3:**
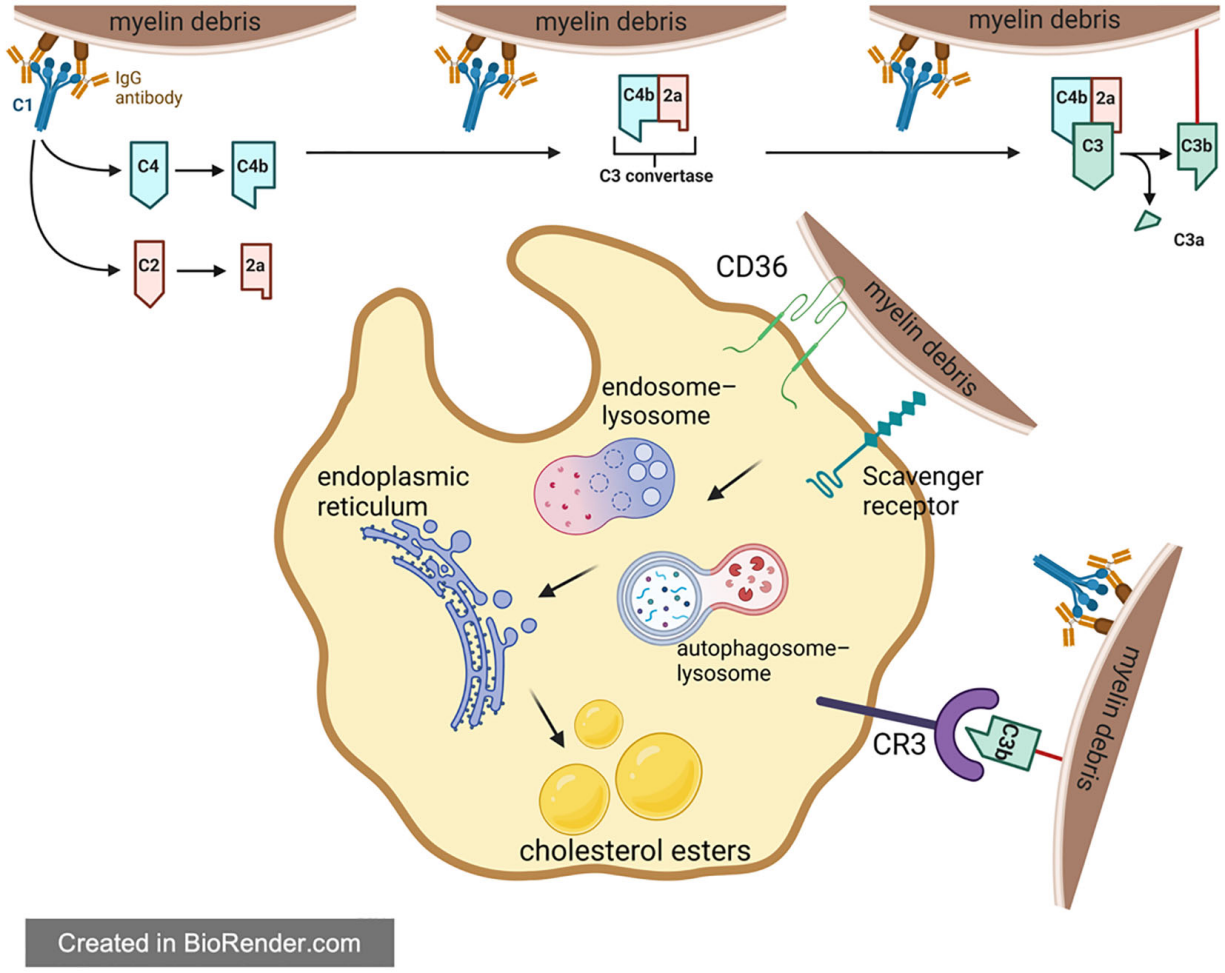
Foam cell formation in spinal cord injury. For one thing, phagocytes directly recognize and engulf myelin debris through a variety of receptors in **Figure 3**. For another thing, complement plays a regulatory role in assisting phagocytes to engulf myelin debris. The phagocytosed myelin debris is first processed by endosome-lysosome and autophagy-lysosome, then reprocessed in the endoplasmic reticulum, and finally transformed into lipid droplets composed of cholesteryl esters in the cell.

A number of studies have clearly shown that foam cells formed after SCI are mainly derived from macrophages, instead of resident microglia and other cells (83–88). As resident immune cells in CNS, microglia are activated immediately upon the injury of spinal cord and play a phagocytic role in the next 3 days. However, with the breakdown of the blood-brain barrier (BBB) and the release of chemokines like CCL2 and CCL3 at the injury site, lots of blood-derived macrophages will gather at the injury center. These macrophages continue to perform phagocytosis through surface receptors shared with microglia, such as scavenger receptors (SR) and trigger receptors expressed on myeloid cell 2 (TREM2) (8, 89, 90). After entering macrophages, these cholesterol-rich myelin debris are generally processed by autophagy-lysosome or endosome-lysosome pathways. With the aging process, myelin debris tends to be metabolized by autophagy-lysosome pathway. However, regardless of the degradation pathway, these degradation products will be transported to the endoplasmic reticulum for reprocessing, and eventually form cholesterol esters, which are stored in the macrophage cell in the form of lipid droplets (**Figure 3**) (82, 91, 92). In response, macrophages will spontaneously excrete these lipids to relieve intracellular lipid stress and avoid negative effects. But in the pathological process of SCI, the lipid processing capacity of macrophages are impaired, leading to intracellular lipid overload. Therefore, macrophages that engulf myelin debris begin to exhibit morphological and functional characteristics similar to foam cells in atherosclerosis at the 7th day of SCI, which are thus called foam cells (93–96).

#### 3.1.2 Influence of foam cells on pathophysiological development of SCI

Following the identification of the mechanism of foam cell formation in SCI, it becomes critical to further explore how they affect the pathophysiological microenvironment at the injury site. It is well known that secondary SCI can be divided into four stages: acute (0-48h), subacute (2-13d), intermediate (2-6w), and chronic (> 6w). Given that the foam cells mentioned above begin to appear at the 7th of SCI, it is reasonable to assume that these cells mainly affect the subacute and subsequent stages (97).

##### 3.1.2.1 Enhancing local inflammation

Several experiments show that, during the transformation of macrophages into foam cells, these macrophages gradually skew to M1 phenotype, releasing inflammatory factors and tilting toward pro-inflammatory. Macrophages can be roughly divided into two phenotypes – M1 and M2 macrophages (98). M1 macrophages are mainly pro-inflammatory and can secrete a variety of pro-inflammatory factors such as tumor necrosis factor-a (TNF-a). This kind of macrophages can be identified by inducible nitric oxide synthase (iNOS) and CD16 et al. Moreover, M2 macrophages are mainly anti-inflammatory, secreting interleukin-10 (IL-10) et al. CD206 and arginase-1 (Arg-1) are the recognition markers of M2 macrophages. The ratio of M1/M2 macrophages greatly affects the pathological state of the injured spinal cord (99, 100). In the natural course of SCI, M2 macrophages began to appear at the 3rd day of injury and reached a peak at the 7th day but they also began to disappear. However, M1 macrophages appeared and reached a peak within one week after the injury and existed for a long time (101). Wang et al. found that macrophages which pre-differentiated from M0 to M2 *in vitro* gradually lost their M2 surface markers after phagocytosis of myelin debris, accompanied by a significant increase in M1 markers, indicating that foam cells were more inclined to exhibit M1 phenotype, namely to exhibit pro-inflammatory property. The team also found that after treatment of macrophages with myelin debris, the activity of STAT3 and STAT6 signaling pathways was suppressed, while the phosphorylation level of intracellular NF-κB inhibitory protein (IκB-α) was up-regulated, thus allowing the release of NF-κB into the nucleus for transcriptional activation (96, 102). In conclusion, macrophages gradually exhibit the characteristics of M1 macrophages during uptake of myelin debris and transformation into foam cells. In other words, foam cells favor pro-inflammatory effects.

Concerning the proinflammatory mechanism of foam cells, the following reasons are as follows: (1) Cholesterol has been found to be transformed into cholesterol crystals after entering the lysosome, causing lysosomal membrane damage and oxidative stress et al., which further activates inflammasome 3 (NLRP3) and finally stimulates inflammation (97, 103). (2) The accumulation of intracellular cholesterol increases the number of lipid rafts on the cell membranes, which enhances the sensitivity of toll-like receptor 4 (Toll4) activated by myeloid differentiation factor 88 (MyD88) to its ligand and amplifies the activation effect of inflammatory factors (104–106). When the formation of foam cells starts, SCI tends to last for 7d which is also the point that M2 macrophages decrease and M1 macrophages dominate. The present authors speculated that the transformation of macrophages into foam cells may be one of the key reasons for the decrease of M2 type macrophages in SCI. However, further research is still needed to verify the hypothesis; the illustration helps uncover the reason why M1/M2 macrophage imbalance in SCI.

##### 3.1.2.2 Decreased phagocytosis of apoptotic cells

After SCI, due to severe inflammatory storm, there remain abundant apoptotic and necrotic cells in the injured area. For various harmful substances released by these dead cells, it is greatly important for SCI recovery to remove the dead cells timely (97). However, the phagocytic effect of foamy macrophages on dead cells is not ideal. As innate immune cells, neutrophils rapidly accumulate in the injured spinal cord under the effects of chemokines, reaching the peak 24 hours after injury and remaining at the injury site for several months (107). Although neutrophils have a certain phagocytosis, helping to purify the microenvironment, their apoptosis and necrosis can still lead to the release of inflammatory factors, chemokines and various proteases, which aggravate tissue edema and necrosis, as well as apoptosis of neurons and oligodendrocytes (108, 109). Therefore, the chemotaxis of neutrophils is negative on SCI repair totally, and thus their timely clearance by macrophages is required. Wang et al. incubated apoptotic neutrophils with foam cells, M1 macrophages and M2 macrophages respectively, and then observed the number of remaining apoptotic neutrophils. The results showed that compared with the number of M1 and M2 macrophages, the number of remaining apoptotic neutrophils in the foam cells group was significantly higher. The finding suggests that the phagocytosis ability of foam cells to apoptotic cells is decreased. However, when the team studied the phagocytosis of foam cells on other non-cellular substances, they found that the phagocytosis of foam cells on ox-LDL, latex beads and yeast polysaccharides was not affected. Therefore, they hypothesized that the phagocytosis of foam cells decreased only for apoptotic cells, while the phagocytosis of other non-cellular materials remained normal (88).

##### 3.1.2.3 Influence nerve regeneration and tissue repair

One of the key points of nerve regeneration after SCI is whether endogenous neural stem cells (NSC) can differentiate into neurons at the injury site and then promote the reconnection of neural circuits. With reference to the factors influencing NSC differentiation, the present authors assume that foam cells may regulate NSC differentiation through the polarization of macrophages. Several researches have shown that in the inflammatory environment caused by M1 macrophages, the number of NSCs that differentiate into astrocytes increases significantly. However, in the anti-inflammatory environment full of M2 macrophages, NSCs mainly differentiate into neurons and oligodendrocytes. Given that foam cells exhibit similar characteristics to M1 macrophages in most cases, the present authors speculate that foam cells may impede the effective differentiation of NSC (110), which should be confirmed by more direct evidence to be found in the future.

In addition, Wang et al. also revealed that foam cells are cytotoxic. They cultured OPCs with medium deriving from M2 macrophages and foam cells for 48h respectively, and then measured the number of live OPCs to assess cytotoxicity. The results showed that the survival rate of OPCs in the foam cells conditioned medium was significantly lower than that in others, and the survival rate of the M2 macrophages group was the highest. This suggests that foam cells have an adverse effect on OPC, which may hinder axon remyelination. After conducting cell scratch experiments on three groups, the team also found that macrophages in foam cells group had less motor ability as well as the poorer healing of the scratch. In contrast, the control group and the M2 macrophages group showed better motor ability and scratch repair (88). These studies all support previous findings that macrophages progressively lose their ability to promote damage repair as they develop into foam cells.

As mentioned before, SCI repair can be hindered by the presence of scar, and studies have shown that foam cells or myelin debris are closely related to scar. Zheng et al. found that 7-day-old mice had no myelin sheath and showed scarless healing after SCI. In order to further verify the relationship between myelin sheath and scar, they added myelin debris or macrophages that had engulfed myelin debris in 3-old-year mice. The result showed that scar appeared in newborn mice that had healed without scar. In addition, the team also demonstrated that effective cholesterol clearance was beneficial in alleviating fibrosis (111). This pro-fibrotic effect of foam cells may be related to the upregulation of intracellular pro-fibrotic cytokines, such as TGF-β [one of astrocyte chemotactic factor (112)], platelet growth factor, and pro-scar transcription factors, such as FOS and JUN (113).

In summary, microglia, neutrophils and macrophages have a series of negative effects on tissue repair and nerve regeneration after the phagocytosis of myelin debris in SCI. Among these cells, foam cells derived from macrophages are particularly critical. Therefore, the reduction of foam cells is essential to promote SCI recovery.

#### 3.1.3 Measures to reduce foam cells

**Figure 4:**
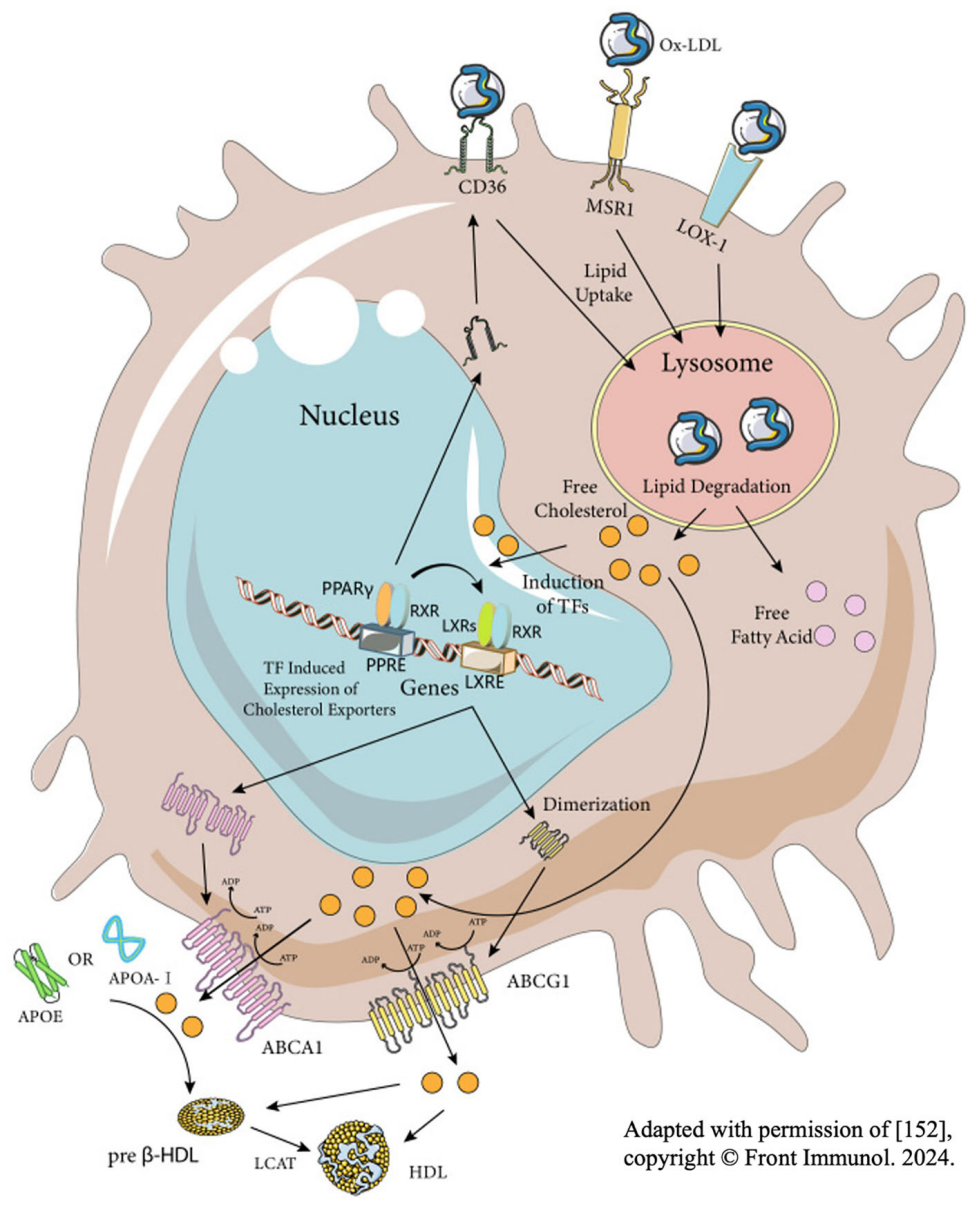
Three key stages of the formation of foam cells–the process of macrophages engulfing, decomposing and exporting lipid. Adapted with permission of (1), copyright ^©^ Front Immunol. 2024.

There are three factors influencing the formation of macrophages into foam cells: the phagocytosis of myelin debris, decomposition of intracellular cholesterol esters and the efflux of intracellular cholesterol ester (**Figure 4**). The irregularity of any step will lead to the existence of many foam cells. Based on existing researches, the interventions can be suggested to be made from the following aspects:

(1) Reduce foam cells at the source: Aiming to the phagocytosis of myelin debris, some scholars blocked its phagocytosis receptors such as CD36 or MSR1, which reduced foam cells and promoted the recovery of SCI (8, 81, 95, 114). However, it is worth considering that such an intervention, while ultimately achieving desirable results, ignores the biological toxicity of myelin debris. Compared with foam cells, myelin debris may play a smaller role in promoting the secondary injury of SCI, so these studies suggest that blocking these phagocytic receptors ultimately shows good SCI repair. However, up to now, it has not been clarified about relative participation of myelin debris and foam cells in promoting the secondary injury of SCI, but this aspect of clarification benefits the research on SCI recovery.
(2) Promote the apoptosis of formed foam cells: Logically, timely apoptosis of foam cells can alleviate a series of negative effects. However, Zheng et al. found that foam cells up-regulated the expression of anti-apoptosis gene CD5L through comparing the transcriptional profile of macrophages before and after they became foam cells, which indicated that foam cells can exist in the injured area for a long time, constantly amplifying inflammation. To further verify this finding, the team transfected a CD5L-targeted SiRNA (SiCD5L) into macrophages. After the co-culture with myelin debris, the microenvironment of the complex was found that the apoptosis inhibition of foam cells got reversed, and the number of foam cells in SCI was reduced accordingly with promising motor function (111).
(3) Promote the efflux of excessive cholesterol in foam cells: Reviewing all the strategies to promote the excretion of intracellular cholesterol, the present authors found a very important axis – PPAR-γ/LXR1/ABCA1. Stimulation of this axis can increase the amount of cholesterol transporter protein ABCA1 on the membrane of macrophages, and more intracellular cholesterol will be released to outside. And foam cells in SCI can be reduced by substances such as adiponectin, D-4F and atorvastatin through this axis (115–119). Like ABCA1, ABCG1 is also a downstream factor of PPAR-γ, mediating cholesterol efflux. However, Zhou et al. found that the experiment results only showed an increase in the number of ABCA1, while the number of ABCG1 changed little after activating the PPAR-γ/LXR1 axis through adiponectin. Therefore, they believed that ABCG1 had little role in maintaining lipid homeostasis (117). However, some scholars have also found that the effect of only increasing the lipid efflux receptor is not satisfied, which needs the aid of extracellular apolipoproteins, i.e. the cholesterol reverse transport system (RCT). And they also pointed out that the foam cells in PNS will disappear in a short time because of the RCT (111). From the perspective of the present authors, Zhou et al. did not take the existing apolipoprotein E (ApoE) in the microenvironment of SCI into consideration, so the result showed that with the increase of ABCA1, foam cells reduced correspondingly. Overall, the present authors believed that the effects of cholesterol transporters and extracellular apolipoproteins are equally important, and both contribution to cholesterol efflux should be considered.
(4) Improve the ability of macrophages to process intracellular lipids. Lipohagy is a kind of selective autophagy for the object of autophagy is lipid. Since Singh first discovered lipohagy in hepatocytes, many researchers have introduced the concept into SCI and identified some related key genes and pathways, providing new insights for the recovery of SCI (120–122). For example, Ryan et al. found that PI3K/AKT/mTOR is up regulated after SCI, and the autophagy ability of macrophages is reduced accordingly, contributing to the existence of foam cells and inflammation. Therefore, by using PI3K/AKT/mTOR inhibitors, the team found that the autophagy of macrophages was restored and foam cells in SCI was reduced correspondingly. Moreover, the inflammation was alleviated and the motor function of mice was well recovered (123). As for the change of the autophagy ability of macrophages, it is acknowledged that it starts to downregulate at the 3rd day of SCI, which coincides with the time when macrophages begin to engulf myelin debris, but the reason for the downregulation is still unclear. In general, it is worthy of further investigation whether or not phagocytosis of myelin debris by macrophages reduces their lipid phagocytosis, which is beneficial to guide the intervention of foam cells (124).

### 3.2 Non-professional phagocytes

In SCI, non- professional phagocytes, such as astrocytes and vascular endothelial cells, can also engulf myelin debris. Although the phagocytic capacity of these cells is not comparable to that of professional phagocytes, the phagocytic capacity of professional phagocytes such as microglia gradually weakens over time. Therefore, the effect of non-professional phagocytes in SCI is also of great significance.

#### 3.2.1 Astrocytes

Several experimental results show that astrocytes participate in the phagocytosis of myelin debris in SCI, and this kind of phagocytosis has certain characteristics: Compared with professional phagocytes that engulf myelin debris in the core area of injury at the early stage, astrocytes are activated and migrate to the peripheral area of injury to engulf myelin debris, the phagocytosis of which is later than that of professional phagocytes (125). Some progress has also been made in the phagocytosis mechanism of astrocytes. At present, many scholars believe that receptors such as LRP1, ABCA1, ABCA7, MEGF10 and GULP1 are closely related to the phagocytosis of myelin debris by astrocytes. Moreover, the increased expression of ABCA1/7 can stimulate the up regulation of MEGF10 and GULP1 (104, 126–130). In SCI, astrocytes usually undergo phenotypic and functional changes, known as reactive astrocytes which can be roughly divided into two categories: A1 and A2. A1 astrocytes are neurotoxic and can hinder the SCI repair, while A2 astrocytes are neuroprotective and can promote the repair (131). After phagocytosis of myelin debris, astrocytes tend to polarize into A1 phenotype, which is detrimental to the recovery of SCI. Meanwhile, the proliferation capacity of astrocytes was up regulated after astrocytes engulf myelin debris, resulting in excessive generation of glial scars that further hinder nerve growth (132).

#### 3.2.2 Vascular endothelial cells

Previous studies have revealed the phagocytosis of vascular endothelial cells. In atherosclerosis, vascular endothelial cells cooperate with macrophages to engulf ox-LDL and jointly participate in the formation of foam cells (133). In SCI, a large number of new microvessels composed of vascular endothelial cells began to appear at the injury site at the 3rd day of SCI. Although these endothelial cells participate in the phagocytosis of myelin debris, they rarely exhibit a foam-like shape (134). What’s more, no literature has been found to indicate that these endothelial cells develop into foam cells in SCI. Zhou et al. found that after SCI, neovascular endothelial cells engulf myelin debris mediated by immunoglobulin IgG. Although the phagocytosis of the vascular endothelial cells is only 1/8 of that of macrophages, the engulfed myelin debris will be further degraded through intracellular autophagy-lysosome pathway. Therefore, the present authors assume that this weak phagocytosis and self-degradation may be one of the reasons why endothelial cells do not develop into foam cells. Although there is no evidence proving that foam cells participate in this section, the team also found that the phagocytosis of myelin debris from endothelial cells also has a series of negative effects, such as increasing macrophage recruitment, increasing pathological angiogenesis, exacerbating local inflammation and demyelination. In addition, endothelial cells may also contribute to the formation of fibrous scars through the transformation into mesenchymal cells (135).

#### 3.2.3 Oligodendrocyte precursor cells (OPCs)

In addition to the amateur phagocytes above, which have been extensively investigated, OPCs has attracted the attention of researchers as a phagocyte homologous to astrocytes. Regarding the mechanism of OPCs processing myelin debris, some teams pointed out that Low-density lipoprotein receptor (LDLR) may be a key element in the successful phagocytosis of myelin debris by OPCs. When dealing with myelin debris, OPCs exhibit similarly to macrophages. Once the intracellular load of myelin debris reaches its maximum, the metabolism of OPCs will be unbalanced, affecting their further differentiation. However, the other effects of OPCs engulfing myelin debris are still unknown, especially the effect on the inflammatory storm after SCI. Further studies of these will help researchers deepen the comprehension of the pathological process of SCI (82, 136).

### 3.3 Complement and immune system

Existing studies have shown that in addition to direct phagocytosis mediated by phagocytic receptor, complement opsonophagocytosis also connects myelin debris and inflammation. The complement system can mark the damage related molecular pattern, and pathogen related molecular pattern et al., supplementing and assisting innate immunity system, so it is called the complement system. However, in this process, inflammatory factors are inevitably produced (87, 137). A large number of complement proteins appeared at the injury site at the 1st day of SCI and persist until chronic stage (138, 139). The complement proteins mainly originate from two aspects: (1) exogenous complement proteins: Exogenous complement proteins are mostly produced in the liver and then released into the blood in a non-activated state. Due to the breakdown of BBB after SCI, these complement proteins are released into CNS. (2) endogenous complement proteins: Another source of complement is *in-situ* cells in spinal cord, including glial cells, neurons, macrophages/microglia et al. Both endogenous and exogenous complement proteins are activated in three ways: classical pathway, bypass pathway and selective pathway (137).

Among many complement proteins, C1 and C5 play a key role in regulating myelin debris phagocytosis. C1q can recognize and bind to antigen-antibody complexes formed by myelin debris and antibodies targeting myelin oligodendrocyte glycoprotein (MOG-IgG1). The triad then activates C2 and C4 to form the C3 convertase, cleaving C3 into C3a and C3b respectively. Then, C3b bound with myelin debris will bind to CR3 on the surface of macrophages, assisting macrophages’ phagocytosis of myelin debris (8). In the pathological process of SCI, the engulfment of myelin debris like this can also lead to intracellular lipid overload in macrophages (82, 140–142). Besides, the C5-9 complex formed during complement activation can lyse intact oligodendrocyte membranes, leading to secondary demyelination and the death of mature oligodendrocytes (141). C5a can act on mast cells and basophils, resulting in the release of vasoactive substances which then increase the permeability of vascular. In addition, C5a is also a chemokine for macrophages and neutrophils, which can recruit inflammatory cells and enhance the inflammatory cascade at the site of injury (143). Moreover, C5a can act on neurons to induce their apoptosis (144). In conclusion, on one hand, the complement system assists the clearance of myelin debris and reduces its negative effects; on the other hand, the complement also amplifies the inflammation, which is not conducive to SCI recovery. Overall, the current results show that the complement system plays a negative role for SCI recovery.

## 4 Discussion

Thereinbefore, the present authors have discussed in detail the influence of myelin debris on the pathophysiological process of SCI from the perspective of its direct and indirect inhibitory effect. Although much of the inhibitory effect of myelin debris is observed, some ideas remain controversial. Though the way how MAIs induce the collapse of axon growth cones and thus hinder SCI recovery has been discussed, some scholars have proposed different views upon it. First, although MAIs plays an “inhibitory” role, they actually promote axon growth. Geoffrey et al. questioned the claim that myelin debris inhibits axon regeneration for the phenomenon that axons are difficult to regenerate through unmyelinated gray matter. Meanwhile, they proposed a novel idea: astrocytes are the main hindrance in axon growth, and the collapse of axon growth cones caused by myelin debris is not to inhibit axon growth but assist axon growth. This idea is mainly based on the two following reasons: (1) The necessary condition for the growth of axons *in vivo* is that the growth cone must follow the corresponding path, but most studies only co-culture neurons with myelin debris in two-dimension space, and thus the simple confrontation between axons and myelin debris cannot accurately reflect the real complex spatial sequence *in vivo*; (2) The inhibition of branches is important to the long-distance connection of regenerated axons in CNS. The collapse of growth cones observed by most researchers is a manifestation of branch inhibition required for growth cone elongation, which acts like a “cage” that limits the direction of axon growth. Thus, inducing collapse is as important as promoting growth in the process of axon regeneration. Inhibition of the branching growth cone provides repulsive contact guidance which excludes all other movement of the axon growth cone except for forward movement, thereby allowing the main axon growth cone to connect to the distal end along the direction of the myelinated nerves (145, 146). Second, the widely recognized “MAIs” do not inhibit axon growth in fact. As Filbin believed (11), the “MAIs” in previous studies did not have inhibitory effects on axon growth cones. It was the change of neurons (mainly the decrease of intracellular cAMP with maturity) that made axon growth cones sensitive to these “inhibitory substances”. Similarly, several research teams have reached similar results (147, 148). However, the present authors hold that the three views on the “inhibitory effect” of myelin debris should be comprehensively considered, and that the inhibition of MAIs on axon growth cone exists, but it is to assist the growth of axons. After the body maturates, neurons strengthen this directional guidance caused by recognizing myelin debris as “inhibitory substances”. However, the mechanism between “MAIs” and axon growth cones inhibition is still very complex, and scholars in the future are suggested to pay more attention to reducing the interference of *in vitro* simulation of SCI as well as eliminating the error of results caused by spatial structure and delayed healing. The correct understanding of the specific effects of MAIs on axon growth can greatly influence the treatment of myelin debris.

There are also several key points about foam cells that deserve attention. The present authors conclude that there is a contradiction among macrophages, myelin debris and foam cells. That is to say, for myelin debris is biologically toxic, the phagocytosis of it by macrophages can alleviate its inhibition of axon growth theoretically, but in fact, macrophages will form foam cells after phagocytosis, which will amplify inflammation, promote scar formation, and not be conducive to nerve regeneration. Some scholars have achieved SCI repair by increasing the phagocytosis of myelin debris. They may believe that the phagocytosis of myelin debris improves the microenvironment of SCI and is conducive to nerve regeneration (149). Regarding drugs for the treatment of SCI, rapamycin, the mTOR inhibitor, promotes the clearance of myelin debris through immunosuppression and induction of autophagy, reducing phagocytes activation and increasing the number of surviving neurons at the injury site (150–156). Similarly, metformin could upregulate the autophagy of phagocytes to a similar effect as rapamycin (91, 163). In addition, some scholars have pointed out that Claulansine F (Clau F), a carazole alkaloid extracted from natural plants, may also have the ability to promote myelin debris clearance with specific mechanism still unclear (92, 162). However, the present authors hold that it is ill-considered because the negative effects of foam cells formed later also need to be under the evaluation of SCI. The logic of this approach is the opposite of the method mentioned above of blocking phagocytosis receptors of macrophages: The former assumes that myelin debris is more harmful to SCI or they fail to see the pathological effects of subsequent foam cells at all while the latter assumes that foam cells are more harmful. Nevertheless, both ideas on SCI repair, the present authors believe, are reasonable explorations, because currently scholars have no deep understanding of myelin debris or foam cells in SCI. The respective roles of both may differ in promoting injury, and it is possible that they play the primary role in promoting injury at different stages. However, further research is still needed on the roles of both in secondary injury of spinal cord injury. Regarding the relationship of macrophages, foam cells, myelin debris and inflammation, another team conversely found that foam cells are originally proinflammatory, but after being stimulated by inflammation, they secrete anti-inflammatory mediators (157, 158). However, inflammation is the main secondary injury after SCI. Inevitably, we have the reason to question whether the anti-inflammatory effects of foam cells can overcome inflammation after SCI. If so, the present authors believe that the presence of foam cells in SCI is reasonable, and instead of trying to reduce foam cells as previous researchers have done, what we need is to reduce their pro-inflammatory effects and enhance their anti-inflammatory ones.

In addition, since myelin debris contains a large number of lipids, which are closely related to iron death, the present authors inevitably question whether the phagocytosis of myelin debris can induce iron death of these phagocytes (159). It has been demonstrated in neurodegenerative diseases that the phagocytosis of myelin debris enhances iron death in cells, but the link between this kind of cell death and myelin debris in SCI is still blank and needs further exploration (160). Similarly, the following questions need to be explored and solved: the difference between foam cells in CNS and PNS; the specific proinflammatory mechanism of foam cells et al. Myelin debris and foam cells in SCI connect multiple processes such as immune inflammation, nerve regeneration, oxidative stress and lipid metabolism, involving various fields such as pathology, pathophysiology and biochemistry. Further investigation of myelin debris and foam cells will greatly deepen human’s understanding of the pathophysiology of SCI and then provide guidance for the treatment of SCI. Given that foam cells in atherosclerosis and foam cells in SCI have similar effects on the pathological process of corresponding diseases (93), the following studies can seek references in the field of atherosclerosis and optimize the treatment of SCI by combining relevant biomaterials in tissue engineering, in order to maximize the recovery of sensory and/or motor functions.

Moreover, the present authors just provided four strategies to reduce the number of foam cells in SCI, some similar opinions are also given in the latest review in this field (1). At present, the authors hold that restoring the lipid autophagy ability of foam cells seems to be the better solution to reduce foam cells. Previous studies have shown that lipid autophagy can not only enhance the clearance of myelin debris from the injury site, but also decrease the intracellular lipid loading capacity. This pathway can clear myelin debris while avoiding the negative effects of foam cells. In other words, it has convincing efficacy before and after myelin debris is engulfed. As mentioned above, the ability of lipid autophagy of macrophages decreases after engulfment of myelin debris from 3 d after SCI. Therefore, determining the specific time point and mechanism of the decline of lipid autophagy in macrophages needs to be conducted so that researchers can manipulate the specific changes in the lipid autophagy ability of macrophages. For example, researchers can monitor the markers related to lipid autophagy at different times after SCI by gene sequencing, bioinformatics screening, PCR and other approaches to explore the trend of lipid autophagy ability of macrophages. Subsequently, these targets can be modified to restore the original ability of macrophages to lipid autophagy. It should be noted that, first, the reasons for the decline of lipid autophagy are various, and the exploration is still in the preliminary stage. Second, the range of capacities for lipid autophagy needs to be established. Because there is an equilibrium point in the lipid autophagy ability of macrophages, exceeding this equilibrium point may lead to phagocyte dysfunction or even cell death (161). The specific methods are as follows.

First, the formation process of foam cells after SCI can be observed by immunofluorescence (myelin debris related antibody), oil red staining, transmission electron microscopy and other methods. Secondly, Western Blot and immunofluorescence can be used to observe the changes in the level of lipid autophagy in macrophages after SCI. Thirdly, the key receptors of lipid autophagy can be screened by transcriptome sequencing technology. Finally, according to the selected results, the gene knockout experimental animals can be constructed to compare the lipid metabolism after SCI and verify the related nerve repair.

The present authors also explained why it is unnecessary to reduce the number of foam cells in SCI. In conclusion, the cognitive process of foam cells in SCI is currently in its infancy, the improvement of which will help to accurately balance the influence of foam cells and then achieve SCI repair.

## 5 Conclusion

In this paper, the adverse effects of myelin debris on SCI recovery have been reviewed in detail. However, it has been neither fully understood about the known “MAIs”, nor the actual effect of axon inhibition mediated by myelin debris, whether to promote or hinder SCI repair, or to depend on neuronal status, which still needs further study. Moreover, there still exist significant limitations in current studies. To be more specific, most studies mainly rely on vitro experiments to explore the negative effects of myelin debris, and only consider the influence of myelin debris while ignoring other factors in the microenvironment SCI, such as oxidative stress, hemodynamic disturbance, inflammatory storm, scar formation and spatial structure of the spinal cord. These factors will affect the pathophysiological development of SCI to varying degrees. And measuring methods vary from team to team. However, all that may affect the results should be carefully considered.

In general, the adverse effects of myelin debris on SCI are still debated in many aspects and need to be further explored. This aspect of investigation provides new insights for promoting SCI recovery in the future.
